# Methyl 5-*O*-(4-chloro­benzo­yl)-2-de­oxy-3-*O*-methyl­sulfonyl-*threo*-pentofuran­oside

**DOI:** 10.1107/S1600536810007087

**Published:** 2010-03-13

**Authors:** Hongshi Jiang, Hui Li, Hong Sun

**Affiliations:** a Department of Applied Chemistry, Yuncheng University, Yuncheng, Shanxi 044000, People’s Republic of China

## Abstract

In the chiral title compound, C_14_H_17_ClO_7_S, an inter­mediate in the synthesis of the AIDS treatment drug zidovudine, the threose ring adopts an envelope configuration, with the O atom at the flap position.

## Related literature

For general background to the title compound, see: Li & Yan (2009 ▶).
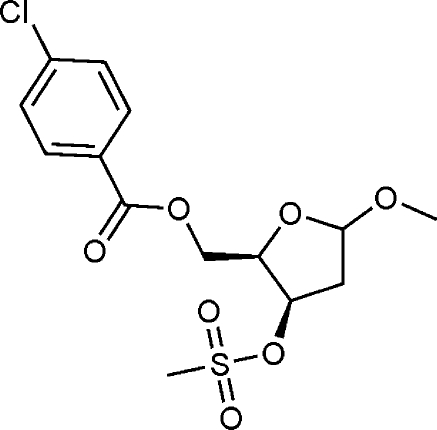


## Experimental

### 

#### Crystal data

C_14_H_17_ClO_7_S
*M* *_r_* = 364.79
Orthorhombic, 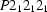

*a* = 5.3103 (11) Å
*b* = 10.996 (2) Å
*c* = 28.559 (6) Å
*V* = 1667.6 (6) Å^3^
*Z* = 4
Mo *K*α radiation
μ = 0.39 mm^−1^
*T* = 113 K
0.16 × 0.04 × 0.02 mm

#### Data collection

Rigaku Saturn CCD diffractometer
Absorption correction: multi-scan (*CrystalClear*; Rigaku/MSC, 2005 ▶) *T*_min_ = 0.941, *T*_max_ = 0.992
10486 measured reflections
3708 independent reflections
2350 reflections with *I* > 2σ(*I*)
*R*_int_ = 0.081

#### Refinement

*R*[*F* ^2^ > 2σ(*F* ^2^)] = 0.061
*wR*(*F* ^2^) = 0.160
*S* = 0.96
3708 reflections
210 parameters
H-atom parameters constrained
Δρ_max_ = 0.39 e Å^−3^
Δρ_min_ = −0.35 e Å^−3^
Absolute structure: Flack (1983 ▶), 1491 Friedel pairs
Flack parameter: 0.14 (12)

### 

Data collection: *CrystalClear* (Rigaku/MSC, 2005 ▶); cell refinement: *CrystalClear*; data reduction: *CrystalClear*; program(s) used to solve structure: *SHELXS97* (Sheldrick, 2008 ▶); program(s) used to refine structure: *SHELXL97* (Sheldrick, 2008 ▶); molecular graphics: *SHELXTL* (Sheldrick, 2008 ▶); software used to prepare material for publication: *CrystalStructure* (Rigaku/MSC, 2005 ▶).

## Supplementary Material

Crystal structure: contains datablocks I, global. DOI: 10.1107/S1600536810007087/hb5317sup1.cif

Structure factors: contains datablocks I. DOI: 10.1107/S1600536810007087/hb5317Isup2.hkl

Additional supplementary materials:  crystallographic information; 3D view; checkCIF report

## Figures and Tables

**Fig. 1.**
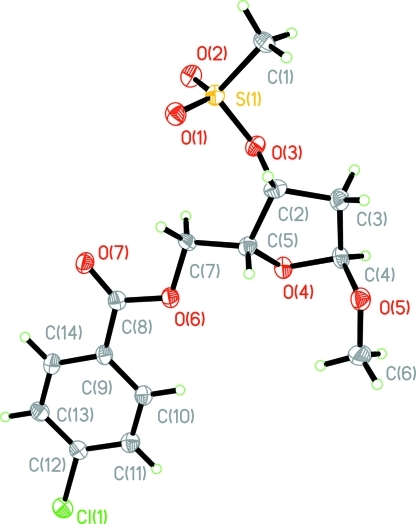
The molecular structure of (I). Displacement ellipsoids are drawn at the 30% probability level and H atoms are shown as small spheres of arbitrary radius.

**Fig. 2.**
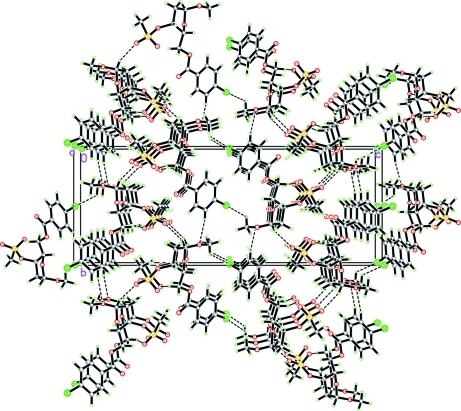
The crystal packing for (I).

## supplementary crystallographic information

### Comment

Zidovudine is a nucleoside analog reverse transcriptase inhibitor, a type of antiretroviral drug. It is the first drug approved for the treatment of AIDS and HIV infection. The structure of the title compound, (I), a key intermediate in the synthesis of zidovudine, is reported here. Single-crystal X-ray diffraction analysis reveals that the title compound crystallizes in the orthorhombic space group *P*2_1_2_1_2_1_.

### Experimental

A solution of methyl sulfonylchloride (21.7 g, 0.19 mol) in dichloromethane (50 ml) was added dropwise to a stirred mixture of methyl 5-*O*-*p*-chloro-benzoyl-2-deoxy-threo-pentofuranoside (45.9 g, 0.16 mol) in triethylamine (30.8 ml, 0.22 mol) at 273-278 K. After stirring for 1 h, ice water (200 ml) was added. After a further 1 h stirring, the organic layer was separated and washed sequentially with hydrochloric acid (5%, 200 ml), saturated aqueous sodium bicarbonate (200 ml) and brine solution (200 ml). The organic extracts were dried with sodium sulphate. The solvent was removed in vacuum and the crude residue purified by flash column chromatography on silica gel. Colourless prisms of (I) were obtained by slow vaporation of a solution in a mixture of ethylacetate and petroleum ether (1:4).

### Refinement

C-bound H atoms were positioned geometrically and refined in the riding-model approximation, with C—H = 0.95-1.00 Å and U_iso_(H) = 1.2 or 1.5U_eq_(C).

### Crystal data

**Table:**

C_14_H_17_ClO_7_S	*F*(000) = 760
*M**_r_* = 364.79	*D*_x_ = 1.453 Mg m^−^^3^
Orthorhombic, *P*2_1_2_1_2_1_	Mo *K*α radiation, λ = 0.71073 Å
Hall symbol: P 2ac 2ab	Cell parameters from 3922 reflections
*a* = 5.3103 (11) Å	θ = 1.4–27.2°
*b* = 10.996 (2) Å	µ = 0.39 mm^−^^1^
*c* = 28.559 (6) Å	*T* = 113 K
*V* = 1667.6 (6) Å^3^	Prism, colorless
*Z* = 4	0.16 × 0.04 × 0.02 mm

### Data collection

**Table:**

Rigaku Saturn CCD diffractometer	3708 independent reflections
Radiation source: rotating anode	2350 reflections with *I* > 2σ(*I*)
multilayer	*R*_int_ = 0.081
Detector resolution: 14.63 pixels mm^-1^	θ_max_ = 27.4°, θ_min_ = 1.4°
ω and φ scans	*h* = −6→6
Absorption correction: multi-scan (*CrystalClear*; Rigaku/MSC, 2005)	*k* = −14→12
*T*_min_ = 0.941, *T*_max_ = 0.992	*l* = −36→36
10486 measured reflections	

### Refinement

**Table:**

Refinement on *F*^2^	Secondary atom site location: difference Fourier map
Least-squares matrix: full	Hydrogen site location: inferred from neighbouring sites
*R*[*F*^2^ > 2σ(*F*^2^)] = 0.061	H-atom parameters constrained
*wR*(*F*^2^) = 0.160	*w* = 1/[σ^2^(*F*_o_^2^) + (0.0761*P*)^2^] where *P* = (*F*_o_^2^ + 2*F*_c_^2^)/3
*S* = 0.96	(Δ/σ)_max_ < 0.001
3708 reflections	Δρ_max_ = 0.39 e Å^−^^3^
210 parameters	Δρ_min_ = −0.35 e Å^−^^3^
0 restraints	Absolute structure: Flack (1983), 1491 Friedel pairs
Primary atom site location: structure-invariant direct methods	Flack parameter: 0.14 (12)

### Special details

**Table:**

Geometry. All esds (except the esd in the dihedral angle between two l.s. planes) are estimated using the full covariance matrix. The cell esds are taken into account individually in the estimation of esds in distances, angles and torsion angles; correlations between esds in cell parameters are only used when they are defined by crystal symmetry. An approximate (isotropic) treatment of cell esds is used for estimating esds involving l.s. planes.
Refinement. Refinement of *F*^2^ against ALL reflections. The weighted *R*-factor wR and goodness of fit *S* are based on *F*^2^, conventional *R*-factors *R* are based on *F*, with *F* set to zero for negative *F*^2^. The threshold expression of *F*^2^ > σ(*F*^2^) is used only for calculating *R*-factors(gt) etc. and is not relevant to the choice of reflections for refinement. *R*-factors based on *F*^2^ are statistically about twice as large as those based on *F*, and *R*- factors based on ALL data will be even larger.

### Fractional atomic coordinates and isotropic or equivalent isotropic displacement parameters (Å^2^)

**Table:**

	*x*	*y*	*z*	*U*_iso_*/*U*_eq_	
S1	1.4386 (2)	0.10450 (10)	0.24656 (4)	0.0343 (3)	
Cl1	−0.0628 (2)	0.49619 (10)	0.49439 (4)	0.0422 (3)	
O1	1.5556 (6)	0.1924 (3)	0.27718 (10)	0.0405 (8)	
O2	1.2922 (6)	0.1486 (3)	0.20756 (10)	0.0425 (8)	
O3	1.2518 (5)	0.0213 (3)	0.27563 (10)	0.0354 (8)	
O4	0.9138 (5)	−0.0285 (3)	0.35800 (10)	0.0344 (7)	
O5	1.0908 (6)	−0.1751 (3)	0.40778 (11)	0.0432 (8)	
O6	0.8593 (6)	0.2219 (3)	0.37504 (10)	0.0372 (8)	
O7	0.8825 (6)	0.4043 (3)	0.33985 (11)	0.0438 (8)	
C1	1.6668 (9)	−0.0004 (4)	0.22686 (16)	0.0409 (11)	
H1A	1.7917	0.0419	0.2076	0.061*	
H1B	1.5848	−0.0639	0.2082	0.061*	
H1C	1.7507	−0.0375	0.2539	0.061*	
C2	1.3140 (8)	−0.0092 (4)	0.32470 (14)	0.0364 (11)	
H2	1.4918	0.0121	0.3327	0.044*	
C3	1.2591 (9)	−0.1449 (4)	0.33224 (17)	0.0442 (13)	
H3A	1.2336	−0.1866	0.3019	0.053*	
H3B	1.4002	−0.1846	0.3490	0.053*	
C4	1.0188 (8)	−0.1493 (4)	0.36168 (15)	0.0369 (11)	
H4	0.8989	−0.2119	0.3495	0.044*	
C5	1.1255 (8)	0.0530 (4)	0.35708 (16)	0.0344 (10)	
H5	1.1994	0.0578	0.3892	0.041*	
C6	0.8817 (12)	−0.1738 (5)	0.43964 (17)	0.0660 (18)	
H6A	0.8109	−0.0916	0.4411	0.099*	
H6B	0.9391	−0.1983	0.4709	0.099*	
H6C	0.7524	−0.2306	0.4287	0.099*	
C7	1.0480 (9)	0.1795 (4)	0.34171 (15)	0.0345 (10)	
H7A	1.1953	0.2347	0.3417	0.041*	
H7B	0.9765	0.1772	0.3097	0.041*	
C8	0.7876 (9)	0.3383 (4)	0.36835 (15)	0.0333 (10)	
C9	0.5748 (9)	0.3752 (4)	0.40003 (14)	0.0341 (10)	
C10	0.4615 (9)	0.2920 (4)	0.43100 (14)	0.0347 (10)	
H10	0.5206	0.2106	0.4324	0.042*	
C11	0.2639 (9)	0.3286 (5)	0.45937 (15)	0.0398 (12)	
H11	0.1832	0.2722	0.4796	0.048*	
C12	0.1858 (9)	0.4488 (4)	0.45785 (15)	0.0349 (10)	
C13	0.2946 (9)	0.5324 (5)	0.42730 (15)	0.0389 (11)	
H13	0.2350	0.6138	0.4260	0.047*	
C14	0.4913 (9)	0.4948 (4)	0.39879 (15)	0.0392 (11)	
H14	0.5695	0.5514	0.3783	0.047*	

### Atomic displacement parameters (Å^2^)

**Table:**

	*U*^11^	*U*^22^	*U*^33^	*U*^12^	*U*^13^	*U*^23^
S1	0.0263 (5)	0.0336 (6)	0.0429 (6)	−0.0003 (5)	−0.0009 (5)	0.0002 (5)
Cl1	0.0340 (6)	0.0454 (7)	0.0473 (6)	0.0034 (6)	0.0002 (5)	−0.0041 (5)
O1	0.0359 (17)	0.0371 (18)	0.0485 (18)	−0.0078 (16)	−0.0019 (16)	−0.0060 (15)
O2	0.0409 (19)	0.045 (2)	0.0421 (19)	−0.0024 (17)	−0.0071 (15)	0.0032 (15)
O3	0.0274 (17)	0.0376 (19)	0.0413 (17)	−0.0057 (14)	−0.0010 (13)	0.0050 (14)
O4	0.0238 (15)	0.0308 (17)	0.0485 (18)	−0.0014 (14)	−0.0039 (14)	0.0003 (13)
O5	0.0414 (19)	0.0372 (19)	0.0511 (19)	0.0062 (17)	0.0016 (16)	0.0043 (14)
O6	0.0388 (19)	0.0297 (17)	0.0431 (18)	0.0019 (14)	0.0032 (13)	−0.0001 (13)
O7	0.0443 (19)	0.0324 (18)	0.055 (2)	0.0036 (16)	0.0098 (16)	0.0077 (15)
C1	0.034 (2)	0.043 (3)	0.046 (3)	0.009 (2)	0.003 (2)	−0.003 (2)
C2	0.023 (2)	0.052 (3)	0.035 (2)	0.005 (2)	−0.0005 (18)	0.007 (2)
C3	0.040 (3)	0.038 (3)	0.055 (3)	0.009 (2)	0.006 (2)	0.008 (2)
C4	0.033 (3)	0.029 (2)	0.048 (3)	0.006 (2)	−0.003 (2)	0.0000 (19)
C5	0.028 (2)	0.033 (2)	0.042 (3)	−0.0045 (19)	0.0035 (19)	0.0022 (19)
C6	0.077 (4)	0.062 (4)	0.059 (3)	0.029 (3)	0.031 (3)	0.015 (3)
C7	0.034 (2)	0.030 (2)	0.039 (2)	0.002 (2)	0.008 (2)	−0.0056 (18)
C8	0.033 (2)	0.033 (3)	0.033 (2)	0.002 (2)	−0.0060 (19)	0.0012 (19)
C9	0.034 (2)	0.030 (2)	0.038 (2)	0.003 (2)	−0.003 (2)	0.0003 (18)
C10	0.032 (2)	0.033 (2)	0.040 (2)	0.001 (2)	−0.003 (2)	0.0006 (19)
C11	0.034 (3)	0.040 (3)	0.045 (3)	−0.004 (2)	0.000 (2)	0.000 (2)
C12	0.032 (2)	0.036 (3)	0.036 (3)	0.003 (2)	−0.005 (2)	−0.0027 (19)
C13	0.036 (3)	0.039 (3)	0.042 (3)	0.007 (2)	0.000 (2)	−0.003 (2)
C14	0.043 (3)	0.030 (2)	0.044 (3)	−0.001 (2)	−0.004 (2)	0.004 (2)

### Geometric parameters (Å, °)

**Table:**

S1—O2	1.442 (3)	C3—H3B	0.9900
S1—O1	1.444 (3)	C4—H4	1.0000
S1—O3	1.585 (3)	C5—C7	1.516 (6)
S1—C1	1.765 (4)	C5—H5	1.0000
Cl1—C12	1.761 (5)	C6—H6A	0.9800
O3—C2	1.479 (5)	C6—H6B	0.9800
O4—C5	1.438 (5)	C6—H6C	0.9800
O4—C4	1.444 (5)	C7—H7A	0.9900
O5—C4	1.400 (5)	C7—H7B	0.9900
O5—C6	1.435 (6)	C8—C9	1.504 (6)
O6—C8	1.349 (5)	C9—C14	1.388 (6)
O6—C7	1.458 (5)	C9—C10	1.408 (6)
O7—C8	1.201 (5)	C10—C11	1.386 (6)
C1—H1A	0.9800	C10—H10	0.9500
C1—H1B	0.9800	C11—C12	1.386 (6)
C1—H1C	0.9800	C11—H11	0.9500
C2—C5	1.524 (6)	C12—C13	1.393 (6)
C2—C3	1.535 (6)	C13—C14	1.388 (6)
C2—H2	1.0000	C13—H13	0.9500
C3—C4	1.529 (6)	C14—H14	0.9500
C3—H3A	0.9900		
			
O2—S1—O1	118.33 (19)	O4—C5—H5	108.9
O2—S1—O3	105.14 (18)	C7—C5—H5	108.9
O1—S1—O3	109.79 (18)	C2—C5—H5	108.9
O2—S1—C1	110.1 (2)	O5—C6—H6A	109.5
O1—S1—C1	109.6 (2)	O5—C6—H6B	109.5
O3—S1—C1	102.7 (2)	H6A—C6—H6B	109.5
C2—O3—S1	119.2 (3)	O5—C6—H6C	109.5
C5—O4—C4	105.8 (3)	H6A—C6—H6C	109.5
C4—O5—C6	112.5 (4)	H6B—C6—H6C	109.5
C8—O6—C7	113.9 (3)	O6—C7—C5	106.9 (3)
S1—C1—H1A	109.5	O6—C7—H7A	110.3
S1—C1—H1B	109.5	C5—C7—H7A	110.3
H1A—C1—H1B	109.5	O6—C7—H7B	110.3
S1—C1—H1C	109.5	C5—C7—H7B	110.3
H1A—C1—H1C	109.5	H7A—C7—H7B	108.6
H1B—C1—H1C	109.5	O7—C8—O6	123.5 (4)
O3—C2—C5	109.1 (3)	O7—C8—C9	124.0 (4)
O3—C2—C3	108.1 (4)	O6—C8—C9	112.5 (4)
C5—C2—C3	103.1 (3)	C14—C9—C10	119.7 (4)
O3—C2—H2	112.0	C14—C9—C8	118.7 (4)
C5—C2—H2	112.0	C10—C9—C8	121.6 (4)
C3—C2—H2	112.0	C11—C10—C9	120.2 (4)
C4—C3—C2	105.4 (4)	C11—C10—H10	119.9
C4—C3—H3A	110.7	C9—C10—H10	119.9
C2—C3—H3A	110.7	C10—C11—C12	119.0 (4)
C4—C3—H3B	110.7	C10—C11—H11	120.5
C2—C3—H3B	110.7	C12—C11—H11	120.5
H3A—C3—H3B	108.8	C11—C12—C13	121.6 (4)
O5—C4—O4	111.1 (3)	C11—C12—Cl1	119.2 (4)
O5—C4—C3	107.2 (4)	C13—C12—Cl1	119.1 (4)
O4—C4—C3	104.7 (4)	C14—C13—C12	118.9 (4)
O5—C4—H4	111.2	C14—C13—H13	120.5
O4—C4—H4	111.2	C12—C13—H13	120.5
C3—C4—H4	111.2	C13—C14—C9	120.5 (4)
O4—C5—C7	111.4 (4)	C13—C14—H14	119.8
O4—C5—C2	104.2 (3)	C9—C14—H14	119.8
C7—C5—C2	114.5 (4)		
			
O2—S1—O3—C2	−162.0 (3)	C8—O6—C7—C5	174.5 (4)
O1—S1—O3—C2	−33.7 (3)	O4—C5—C7—O6	59.2 (4)
C1—S1—O3—C2	82.8 (3)	C2—C5—C7—O6	177.1 (3)
S1—O3—C2—C5	112.1 (3)	C7—O6—C8—O7	−5.1 (6)
S1—O3—C2—C3	−136.5 (3)	C7—O6—C8—C9	174.2 (3)
O3—C2—C3—C4	−106.4 (4)	O7—C8—C9—C14	−4.3 (7)
C5—C2—C3—C4	9.0 (4)	O6—C8—C9—C14	176.4 (4)
C6—O5—C4—O4	62.4 (5)	O7—C8—C9—C10	176.4 (5)
C6—O5—C4—C3	176.2 (4)	O6—C8—C9—C10	−2.9 (6)
C5—O4—C4—O5	79.2 (4)	C14—C9—C10—C11	1.4 (7)
C5—O4—C4—C3	−36.2 (4)	C8—C9—C10—C11	−179.3 (4)
C2—C3—C4—O5	−102.4 (4)	C9—C10—C11—C12	−2.1 (7)
C2—C3—C4—O4	15.6 (4)	C10—C11—C12—C13	2.4 (7)
C4—O4—C5—C7	166.4 (3)	C10—C11—C12—Cl1	−179.2 (3)
C4—O4—C5—C2	42.4 (4)	C11—C12—C13—C14	−2.0 (7)
O3—C2—C5—O4	84.0 (4)	Cl1—C12—C13—C14	179.6 (3)
C3—C2—C5—O4	−30.7 (4)	C12—C13—C14—C9	1.3 (6)
O3—C2—C5—C7	−37.9 (5)	C10—C9—C14—C13	−1.0 (6)
C3—C2—C5—C7	−152.6 (4)	C8—C9—C14—C13	179.7 (4)
